# Structural Basis of Thermal Stability of the Tungsten Cofactor Synthesis Protein MoaB from *Pyrococcus furiosus*

**DOI:** 10.1371/journal.pone.0086030

**Published:** 2014-01-20

**Authors:** Nastassia Havarushka, Katrin Fischer-Schrader, Tobias Lamkemeyer, Guenter Schwarz

**Affiliations:** 1 Institute of Biochemistry, Department of Chemistry, University of Cologne, Cologne, Germany; 2 Cluster of Excellence in Cellular Stress Responses in Aging-associated Diseases (CECAD), University of Cologne, Cologne, Germany; 3 Center for Molecular Medicine Cologne, University of Cologne, Cologne, Germany; University of Alberta, Canada

## Abstract

Molybdenum and tungsten cofactors share a similar pterin-based scaffold, which hosts an ene-dithiolate function being essential for the coordination of either molybdenum or tungsten. The biosynthesis of both cofactors involves a multistep pathway, which ends with the activation of the metal binding pterin (MPT) by adenylylation before the respective metal is incorporated. In the hyperthermophilic organism *Pyrococcus furiosus*, the hexameric protein MoaB (PfuMoaB) has been shown to catalyse MPT-adenylylation. Here we determined the crystal structure of PfuMoaB at 2.5 Å resolution and identified key residues of α3-helix mediating hexamer formation. Given that PfuMoaB homologues from mesophilic organisms form trimers, we investigated the impact on PfuMoaB hexamerization on thermal stability and activity. Using structure-guided mutagenesis, we successfully disrupted the hexamer interface in PfuMoaB. The resulting PfuMoaB-H3 variant formed monomers, dimers and trimers as determined by size exclusion chromatography. Circular dichroism spectroscopy as well as chemical cross-linking coupled to mass spectrometry confirmed a wild-type-like fold of the protomers as well as inter-subunits contacts. The melting temperature of PfuMoaB-H3 was found to be reduced by more than 15°C as determined by differential scanning calorimetry, thus demonstrating hexamerization as key determinant for PfuMoaB thermal stability. Remarkably, while a loss of activity at temperatures higher than 50°C was observed in the PfuMoaB-H3 variant, at lower temperatures, we determined a significantly increased catalytic activity. The latter suggests a gain in conformational flexibility caused by the disruption of the hexamerization interface.

## Introduction

**Figure 1:**
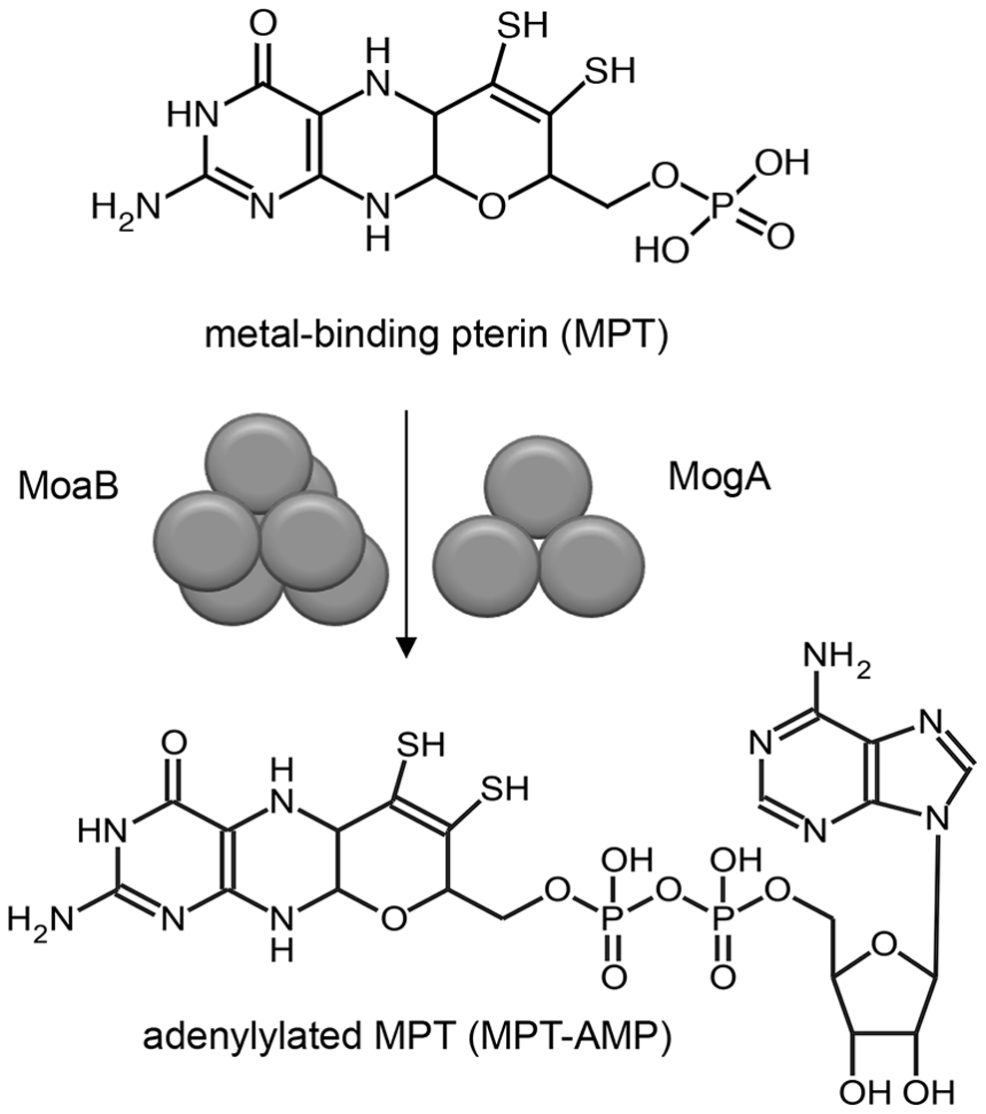
Adenylylation of the cofactor intermediate MPT catalyzed by hexameric MoaB (*P. furiosus*) and trimeric MogA (*E. coli*).

Molybdenum and tungsten enzymes are involved in key reactions in the global cycles of carbon, nitrogen and sulphur [1]. Most members of the family of molybdenum and tungsten enzymes catalyse oxygen transfer reactions [1]–[3]. While tungsten-containing enzymes are restricted to bacteria and archaea with most of them being found in thermophilic and hyperthermophilic archaea, molybdenum-containing enzymes are found in all kingdoms of life [4]. Within their active site, molybdenum or tungsten are chelated by a metal-binding pterin (MPT, also known as molybdopterin) forming the so-called molybdenum (Moco) or tungsten cofactors (Wco), respectively [1]. Wco and Moco biosyntheses represent multistep pathways that start from GTP and involve three isolatable intermediates: cyclic pyranopterin monophosphate (cPMP) [5], MPT and adenylylated MPT (MPT-AMP) [6]. MPT-adenylyl-transferases (EC 2.7.7.75) convert MPT to MPT-AMP (Figure 1), prior to the final metal insertion step, which requires either tungstate or molybdate to produce Wco and Moco, respectively [7], [8].

Biochemical and functional characterization of MPT-adenylyl-transferases from different organisms revealed that they all fold in a similar tertiary structure while presenting two different oligomeric states: trimers (MogA sub-family) and hexamers (MoaB sub-family). Structural analysis of MoaB proteins demonstrated that MoaB hexamers are formed by dimerization of trimers [9], [10]. Catalytically active, trimeric MPT-adenylyl-transferases were identified in mesophilic organisms, *e.g. E. coli* MogA protein, G domain of the plant Cnx1 protein (Cnx1G) and G domain of the human gephyrin protein (GephG) [7], [11], [12]. In contrast, the only characterized hexameric MPT-adenylyl-transferase, MoaB, was purified from the hyperthermophilic archaeon *P. furiosus* and showed maximal activity at 80°C [8], thus being close to the optimal growth temperature of *P. furiosus* of 100°C [13]. Therefore, enzymes of the MPT-adenylyl-transferase family from hyperthermophilic organisms represent a suitable target for the elucidation of the impact of oligomerization on thermal stability.

In this study we investigated the influence of hexamerization on the structural stability and enzymatic activity of *P. furiosus* MPT-adenylyl-transferase MoaB (PfuMoaB). We determined the crystal structure of PfuMoaB at 2.5 Å resolution and engineered a hexamerization-deficient variant of PfuMoaB using structure-guided mutagenesis. The resulting variant was significantly impaired in thermal stability and MPT-adenylylase activity at high temperatures. However, we found a gain of function at low temperatures suggesting that hexamer formation in PfuMoaB is a key factor contributing to thermal stability in PfuMoaB by retaining its structural integrity at high temperatures.

## Materials and Methods

### Protein Expression and Purification

*PfumoaB-WT* and *EcomogA* were cloned into *Bam*HI and *Hind*III restriction sites of pQE80L (Qiaqen) using pET15b_*PfumoaB-WT* and pET22b_*EcomogA* [8] as templates for PCR, resulting in the fusion of an N-terminal His-tag. *PfumoaB-H3* was *in vitro* synthesized by GenScript and cloned into pQE80L in the same way as *PfumoaB-WT*. *E. coli* MPT synthase subunits MoaD and MoaE [14], *E. coli* MoaB, *E. coli* MogA [8] and *A. thaliana* Cnx1G [15] were expressed and purified as previously described. For crystallization, PfuMoaB-WT was purified as described [8]. For all other procedures PfuMoaB-WT and PfuMoaB-H3 were expressed in *E. coli* BL21(DE3) for 16 h at 18°C. Expression was induced with 250 µM IPTG at a cell density of OD_600_ of 0.5. Proteins were purified using ion metal affinity chromatography on Ni^2+^-nitrilotriacetic (Ni-NTA) matrix (Ni-NTA Superflow Cartridge, Qiagen) attached to Äkta Purifier (GE Healthcare) following the manufacturer’s instructions. Purified proteins were exchanged into buffer containing 0.1 M Tris pH 8.0 and 200 mM NaCl using PD10 columns (GE Healthcare), flash frozen in liquid nitrogen and stored at –80°C until further use.

### Protein Crystallization

For crystallization, purified PfuMoaB-WT was exchanged into a buffer containing 20 mM Tris pH 8.0, 100 mM NaCl using PD10 columns (GE Healthcare). Crystallization conditions were tested using screening solutions purchased from Qiagen (The JCSG Core I, II, III and IV Suite). Routinely, 300 nL of 5 mg/ml PfuMoaB protein solution were mixed with 300 nL of screening solution applying a vapour diffusion technique in 96-well Intelli plates (sitting drop). Screens were set up using Hydra II-eDrop robot and ControlMate software (Matrix Technology). Plates were sealed with a clear seal film, centrifuged for 1 min at 1000×g and incubated at 20°C. After one week, protein crystals were observed in the condition containing 0.1 MES pH 6.0, 40% PEG 400 and 5% PEG 3000 (JCSG Core screen III, condition F1). Crystals were extracted directly from the screen condition, mounted into nylon loops (Hampton Research) and flash frozen in liquid nitrogen without addition of cryo-protectant.

### Data Collection and Structure Determination

Diffraction data were collected at the BESSY II beamline BL 14.1 (Berlin, Germany) at a wavelength of 0.918 Å, with 0.5° oscillation range and a crystal to detector distance of 225 mm. Diffraction data were processed with MOSFLM [16] and SCALA of the CCP4 program suite [17]. Data collection statistics are summarized in Table S1.

The crystal structure of PfuMoaB was determined by molecular replacement with PHASER [18] using the coordinates of *Bacillus cereus* MoaB (1Y5E) as a search model. Refinement was performed with REFMAC5 [19] with 5% randomly chosen reflections that were set aside to calculate *R*-free. The model was manually refined using COOT [20]. Water molecules were added with COOT, ligand molecules were included manually. The structure was refined to 2.5 Å resolution with final *R-*work and *R-*free of 0.18 and 0.24, respectively and was validated using COOT validation tools. Structural coordinates were deposited in the RCSB protein data bank (4LHB). Refinement statistics are shown in Table S1.

### Circular Dichroism (CD) Spectroscopy

CD spectra were recorded from 195 to 260 nm in a 0.1 cm light path quartz cuvette at 20°C with a scanning speed of 10 nm/min using a Jasco J-715 CD spectropolarimeter (Jasco). The concentration of the protein samples was adjusted to 0.2 mg/ml in 10 mM sodium phosphate buffer, pH 8.0. Each spectrum was recorded five times and averaged. Averaged CD spectra were corrected by buffer baseline by subtraction. Measured elllipticity θ in milli-degrees was converted into mean residues ellipticity [θ] in deg * cm^2^ * dmol^–1^ using following formula: [θ] = θ * M_r_/(10 * (n−1)*c * L), where M_r_ is the molecular weight of a protein in Da, n is the number of residues per monomer of protein, c is the sample concentration in mg/ml and L is the path length in cm.

### Size Exclusion Chromatography (SEC)

For size exclusion chromatography (SEC) a preparative Superdex 200 16/60 prep grade and an analytical Superdex 200 10/300 column were used (GE Healthcare). Routinely, a SEC buffer containing 0.1 M Tris pH 8.0 and 0.2 M NaCl, was applied. Protein separation was conducted with a flow rate of 0.5 ml/min at 4°C. Absorbance was monitored at 280 nm. Protein-containing fractions derived from the preparative SEC column were collected, concentrated by ultrafiltration (Amicon, Millipore), flash-frozen in liquid nitrogen and stored at −80°C until further use. Molecular masses of proteins were determined using standard proteins purchased from GE Healthcare (Gel Filtration Calibration Kit, HMW).

### Determination of Molecular Mass of PfuMoaB-WT and PfuMoaB-H3 by Mass Spectrometry

Protein samples (100 µl) with a concentration of 2 mg/ml were diluted to a final volume of 450 µl with 20% acetonitrile in water containing 1% acetic acid. Samples were concentrated 10-fold by centrifugation using 3 kDa Amicon ultrafiltration units (Millipore). This buffer exchange was repeated three times. Protein concentration was determined by infrared spectrometry (Direct Detect, Millipore) and adjusted to 2 mg/ml. Electrospray ionization mass spectrometry was performed with a LTQ Orbitrap mass spectrometer (LTQ Orbitrap Discovery, Thermo Scientific). The instrument’s built-in syringe pump delivered the sample at a flow rate of 1 µl/min. In the Orbitrap 100 full scan MS spectra were acquired in the m/z range 150–2,000 at a resolution of 30,000 and averaged. Data acquisition was carried out for 1 min. For charge state deconvolution spectra were exported to the MagTran software (version 1.02, [21]). Protonated (MH+) molecular masses were calculated in the mass range 10,000–30,000 Da.

### Cross-linking

Bis-sulfosuccinimidyl suberate (BS^3^) and 1-Ethyl-3-[3-dimethylaminopropyl]-carbodiimide hydrochloride (EDC) were purchased from Thermo Scientific. 0.5 mg/ml protein solution was incubated with 1.5 mM BS^3^ in a buffer containing 20 mM sodium phosphate pH 8.0 and 0.2 M NaCl for 30 min or with 2 mM EDC in a buffer containing 0.1 M MES pH 6.0 and 0.5 M NaCl for 3 h. Cross-linking reactions were quenched with 50 mM Tris pH 7.5 for 15 min and further subjected to SDS-PAGE. Bands with molecular masses corresponding to PfuMoaB-WT and PfuMoaB-H3 trimers were cut from the SDS polyacrylamide gels, digested with trypsin and analysed by LC-MS/MS in the proteomics facility of the Cologne Cluster of Excellence in Cellular Stress Responses in Aging-associated Diseases (CECAD).

### Tryptic in-gel Digestion and Nano-LC ESI-MS/MS

SDS-PAGE bands of interest were subjected to tryptic in-gel digestion according to the reference [22] with minor modifications. Prior to nano-LC-MS/MS analysis the peptides were desalted using STAGE Tip C18 spin columns (Proxeon/Thermo Scientific) as described elsewhere [23]. Eluted peptides were concentrated *in vacuo* and then re-suspended in 0.5% acetic acid in water. Analyses using reversed phase liquid chromatography coupled to nano-flow electrospray tandem mass spectrometry were carried out using an EASY nLC II nano-LC system (Proxeon/Thermo Scientific) with a 150 mm C18 column (internal diameter 75 µm, Dr. Maisch GmbH) coupled to a LTQ/Orbitrap mass spectrometer (LTQ Orbitrap Discovery, Thermo Scientific). Peptide separation was performed at a flow rate of 250 nl/min. over 79 minutes (5 to 10% acetonitrile in 2 min., 10 to 40% in 60 min., 40 to 100% in 2 min., wash at 100%; buffer A: 0.1% formic acid in H_2_O; buffer B: 0.1% formic acid in acetonitrile). Survey full scan MS spectra (m/z 350 to 2000) of intact peptides were acquired in the Orbitrap at a resolution of 30000 using m/z 445.12003 as a lock mass. The mass spectrometer acquired spectra in „data dependent mode“ and automatically switched between MS and MS/MS acquisition. Signals with unknown charge state and +1 were excluded from fragmentation. The ten most intense peaks were isolated and automatically fragmented in the linear ion trap using collision-induced dissociation (CID). Databases containing cross-linked peptides were generated for PfuMoaB-WT and the H3 variant using the publicly available xComb software (version 1.3) with the following settings: enzyme specificity trypsin, two missed cleavages, intra- and inter-molecular cross-links, type of cross-linker used EDC, minimum peptide length 4 amino acid residues. Sequest as implemented in the Proteome Discoverer 1.3 software (Thermo Scientific) was used for identification by searching the databases generated by the xComb software. Oxidation at methionine residues was used as a variable modification and carbamidomethylation at cysteine residues as fixed modification. No modification was used for the cross-linker EDC. The xComb software computes every possible cross-link combination by linearizing each pair of tryptic peptides. Because of this linearization process, each pair of peptides is assembled in two permutations, i.e. peptide A followed by peptide B and *vice versa*. The resulting xComb database was created from digested peptides, and not from proteins. Therefore, an enzyme mode was generated that cleaves after a non-existing residue (specified as “J” which is not cleaved), to simulate a “do not cleave” mode. Sequest then searches the xComb database without performing any enzymatic digestion and takes each entry in its whole. Mass tolerance for intact peptide masses was 10 ppm for Orbitrap data and 0.8 Da for fragment ions detected the linear trap. Search results were filtered to contain only high confident peptides (false discovery rate ≤1%) with a mass accuracy of ≤5 ppm, and a peptide length of ≥6 amino acid residues. Only peptides for which at least two fragment spectra (peptide spectral match, PSM) were detected were further analyzed. The minimum protein score was set to ≥4.0.

### Differential Scanning Calorimetry (DSC)

DSC was performed using a VP-DSC MicroCalorimeter (MicroCal, GE Healthcare). Protein samples were diluted to the concentration of 50 µM in a buffer containing 50 mM sodium phosphate pH 8.0 and 100 mM NaCl, degased and heated from 20°C to 130°C with a scan rate of 90°C per hour. Melting temperatures (*T*_m_) were determined using ORIGIN 7 software (OriginLab Corporation).

### MPT and MPT-AMP Synthesis *in vitro*

cPMP was purified as previously described [5]. All reactions were conducted in a buffer containing 0.1 M Tris pH 7.2. For MPT synthesis 100 pmol MoaE, 3500 pmol MoaD and 1000 pmol cPMP were incubated at room temperature for 60 min in the presence of 1000 pmol PfuMoaB-WT or PfuMoaB-H3 variant. Next, the reaction mix was treated for 1 min at the respective temperature prior to the initiation of adenylylation with 5 mM ATP and 1 mM MgCl_2_. MPT and MPT-AMP were oxidised to their stable, fluorescent derivates, FormA and FormA-AMP [8], respectively, and quantified by high-pressure liquid chromatography (HPLC).

### Determination of MPT and MPT-AMP Content

Synthesised MPT and MPT-AMP were converted with iodine into their fluorescent derivates FormA and FormA-AMP, respectively, further treated with calf alkaline phosphatase (Roche) to yield FormA-dephospho [8]. FormA-dephospho and FormA-AMP were separated on a HPLC column C4 ReproSil 100, 250 mm x 4.6 mm, 5 µm particle size (Dr. Maisch) attached to an Agilent 1200 HPLC System (GE Healthcare) in 10 mM sodium phosphate buffer pH 3.0 and methanol gradient from 10% to 23% at 2 ml/min flow rate. FormA-dephospho and FormA-AMP were quantified as described [7], [15].

### Bioinformatic Methods

Coordinates of crystal structures of *A. aeolicus* MogA (3MCI) and *T. thermophilus* MogA (3MCH) were extracted from the Protein Data Bank of Research Collaboratory for Structural Bioinformatics (PDB RSCB, http://www.rcsb.org/pdb/home/home.do); protein sequences were from the protein database of the National Center for Biotechnology Information (NCBI, http://www.ncbi.nlm.nih.gov/protein), reference number NP_213032.1 and WP_011174010.1, respectively. Interface analysis of protein crystal structures was performed using the PISA server of the European Bioinformatics Institute (EBI) (http://www.ebi.ac.uk/) [24]. Salt bridge content was determined using the “Protein Interaction Calculator” (PIC) server [25] provided by Indian Institute of Science, Bangalore (http://pic.mbu.iisc.ernet.in/). Hydrogen bond content was determined using the “Hydrogen Bond Calculator” server of the Center for Informational Biology of the University Ochanomizu, Japan (http://cib.cf.ocha.ac.jp/bitool/HBOND/).

## Results

### Determination of the Crystal Structure of PfuMoaB

PfuMoaB was crystallized in the space group P3_1_21 with three molecules (chains A, B and C) in the asymmetric unit. The crystallographic model was refined to a *R*-factor of 0.18 (*R*-free 0.24). The stereo-chemical statistics derived from the final coordinates are summarized in Table S1. The N-terminal His-tag as well as residues 1–10 in chain A, residues 1–11 in chain B and residues 1–9 in chain C were disordered, and are therefore not present in the model. Side chains of Lys12, Lys24, Arg30, Arg87, Glu134 and Glu169 had poor electron density suggesting their high flexibility.

**Figure 2:**
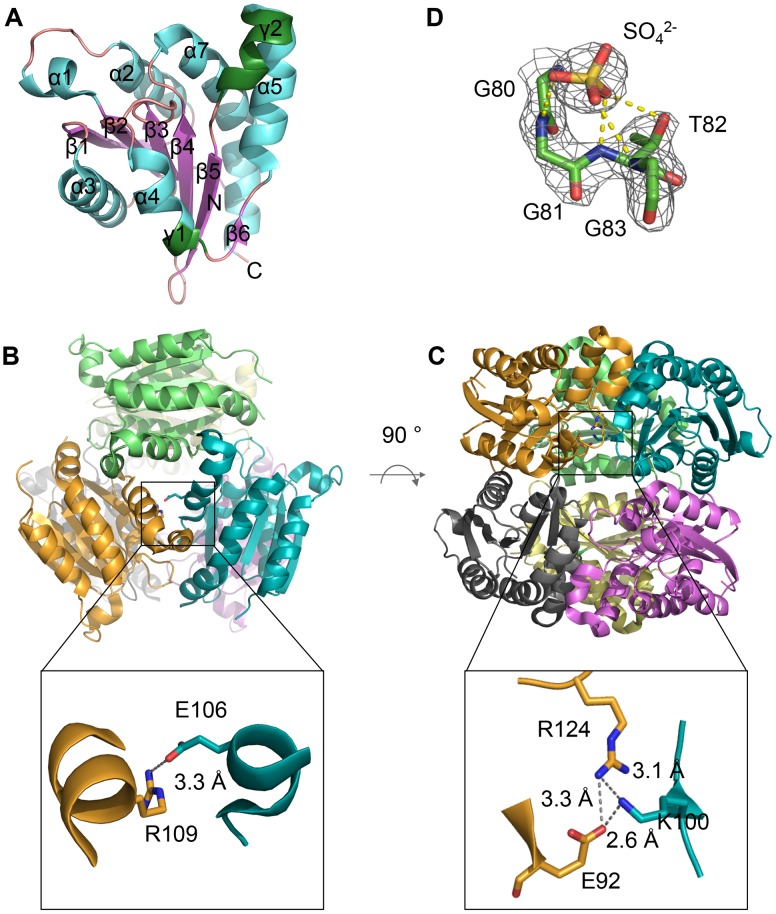
Crystal structure of PfuMoaB. (A) Ribbon representation of PfuMoaB monomer, secondary structure elements, N- and C-termini are labelled; α-helices are coloured in cyan, β-sheets in magenta, 3_10_-helices in green, loops in pink. (B) and (C) top and side view of the PfuMoaB hexamer, respectively. Subunits are shown in different colours. Zoom-in represent ionic interactions at the trimerization interface of PfuMoaB-WT. Residues mediating the contacts between subunits are shown in stick representation and are labelled. (D) Sulfate ion at the active site of PfuMoaB. The residues of the conserved Gly-Gly-Thr-Gly motif and the sulfate ion are shown superimposed with the experimentally phased electron density, contoured at 1 σ.

Each PfuMoaB subunit consists of a slightly bent central β-sheet, surrounded by six α-helices and two 3_10_ helices. The β-sheet comprises six strands: five parallel (β1– β4,) and one antiparallel (β5) embedded between the inner β4 and the flanking β6 strands (Figure 2A). The molecular architecture is consistent with structures of other MPT-adenylyl-transferases. The functional oligomeric state of PfuMoaB was shown to be a hexamer [8]. In the crystal structure a hexamer can be generated by rotation of the asymmetric unit by 180° around the two-fold crystallographic symmetry axis. Therefore, similar to the other proteins of the MoaB family, PfuMoaB represents a dimer of trimers (Figure 2B–C).

The active site of MPT-adenylyl-transferases encompasses a highly conserved Gly-Gly-Thr-Gly motif, which is involved in ATP hydrolysis and the coordination of the resulting MPT-AMP diphosphate [6]. In the crystal structure of PfuMoaB residual electron density was observed in close proximity to the Gly-Gly-Thr-Gly motif. As no co-purified MPT or MPT-AMP was detected in PfuMoaB, the observed electron density was attributed to components of the crystallization solution. In crystal structures of PfuMoaB homologues, *E. coli* MogA and *E. coli* MoaB, a sulfate anion was detected in the same position close to the Gly-Gly-Thr-Gly motif, most probably mimicking the phosphate of MPT [26], [27]. PfuMoaB crystals were grown in a solution containing 2-(N-morpholino)ethanesulfonic acid (MES), which with its negatively charged sulfonate part resembles sulfate or phosphate. This suggests a binding of the MES molecule into the active site of PfuMoaB via the sulfonate. The MES molecule was manually modelled into the additional density, resulting in a good fit for the sulfonate part, but high B-factors for the MES morpholino ring, implying a high flexibility of the latter. Therefore, only a sulfate molecule was manually positioned into the respective electron density patch (Figure 2D) leading to an improved overall model quality.

### The PfuMoaB Hexamer

The surface of each PfuMoaB monomer is involved in the organization of two interfaces: the hexameric and trimeric contact sides, respectively. At the interface between the subunits of the trimer the γ2-helix of one subunit forms a prominent bulge that intrudes into the surface between α5- and α7-helices of the adjacent monomer. Furthermore, the loop connecting β3 with α4 provides a significant contact area with C-terminal residues of α7-helix of the neighbouring subunit within the trimer. Assembly of subunits into trimers is additionally strengthened through several ionic interactions, including salt bridges between Arg109 of one monomer and Glu106 of another monomer (Figure 2B), and a cluster of ionic interactions involving Lys100 of one subunit and Arg124 and Glu92 of the adjacent polypeptide chain (Figure 2C).

**Figure 3:**
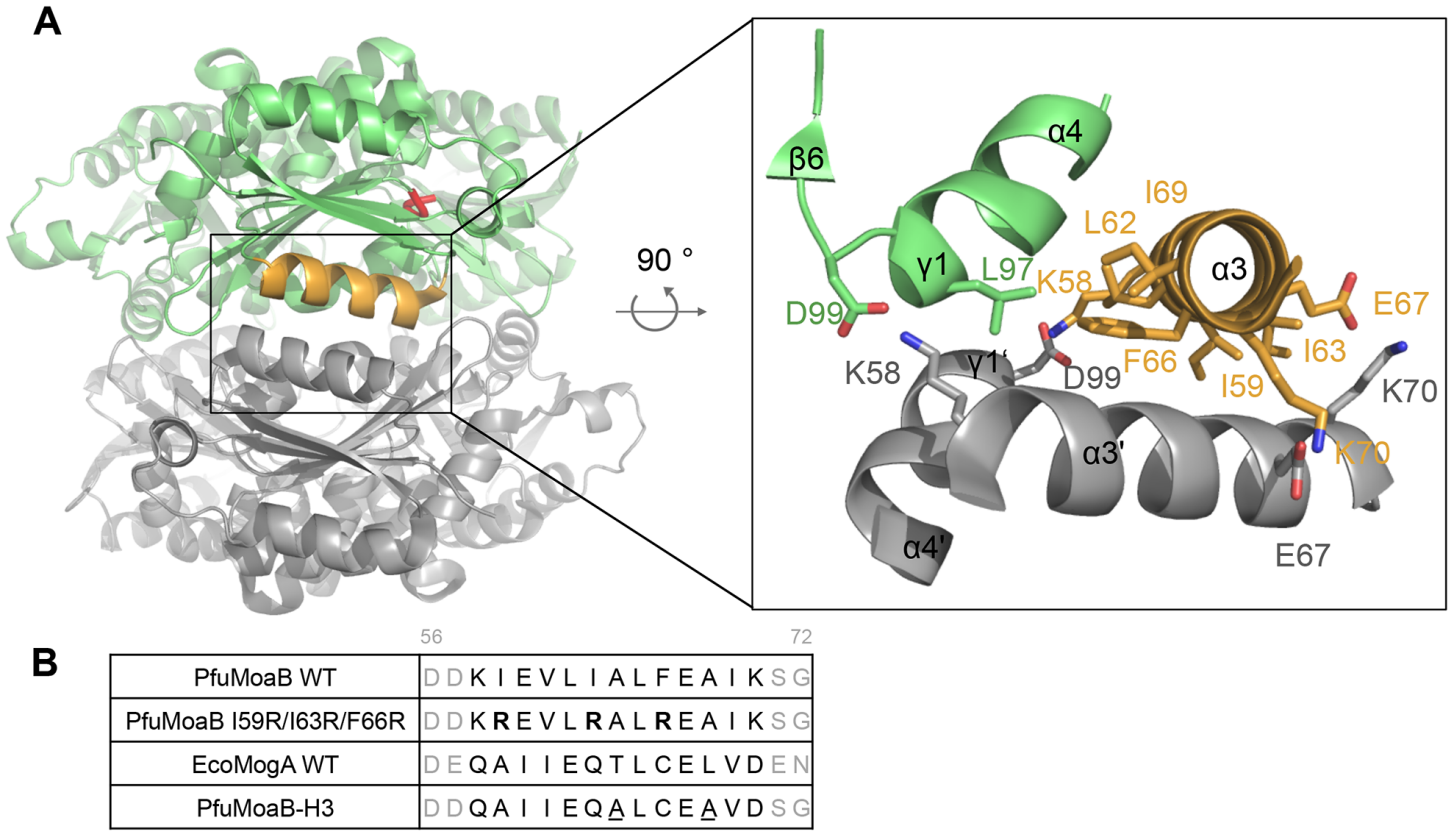
Hexamerization interface and structure-guided mutagenesis of PfuMoaB. (A) Side view of PfuMoaB hexamer. Two trimers of PfuMoaB are depicted in grey and green. The conserved Gly-Gly-Thr-Gly motif within the active site is shown in the upper PfuMoaB monomer in red. The trimer-trimer interaction interface is boxed-in. α3-helix of the upper monomer is depicted in orange. The zoom-in shows labelled residues at the interface in stick representation. As the interface is build up by identical surfaces of each subunit, the hydrophobic residues of the bottom subunit (Ile59, Leu62, Ile63, Phe66, Ile69, Leu97) are not shown for the sake of clarity. (B) Structure-guided mutagenesis of PfuMoaB. Sequences of α3-helix of PfuMoaB-WT and EcoMogA are coloured in black, flanking residues of the helices are represented in grey. Residues are numbered accordingly to the PfuMoaB sequence. Sequences of the variants PfuMoaB I59R/I63R/F66R and PfuMoaB-H3 are shown. Arginine residues of PfuMoaB I59R/I63R/F66R are depicted in bold. Residues of PfuMoaB-H3, which were not exchanged accordingly to the EcoMogA sequence, are underlined.

**Figure 4:**
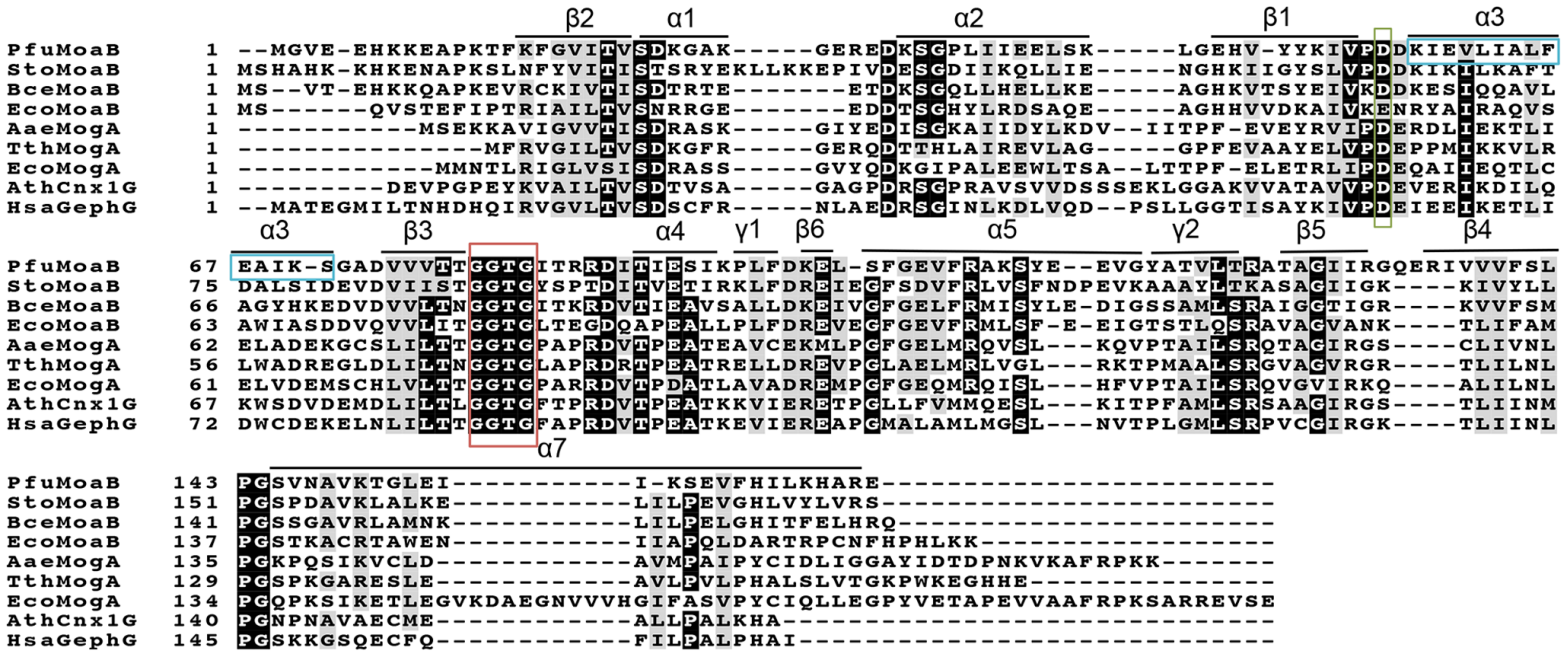
Multiple sequence alignment of MPT-adenylyl-transferases from different organisms. Corresponding MPT-adenylyl-transferases are abbreviated as follows: PfuMoaB, *Pyrococcus furious*; StoMoaB, *Sulfolobus tokodaii*; BceMoaB, *Bacillus cereus;* EcoMogA and EcoMoaB, *Escherichia coli*; TthMogA, *Thermus thermophilus*; AaeMogA, *Aquifex aeolicus*; *Arabidopsis thaliana*; HsaGephG, *Homo sapiens*. Secondary structure elements of PfuMoaB are shown. The conserved MPT-binding motif GGTG is highlighted with a red box, the conserved aspartate residue coordinating Mg^2+^- ion with a green box [6], residues of PfuMoaB α3-helix with a blue box. Highly conserved residues are depicted in white letters and black background; semi-conserved residues are shadowed in grey. Consensus threshold was set to 0.8. Sequences were aligned with Clustal Omega [62],and modified with BoxShade server (Swiss Institute of Bioinformatics).

Interactions between two trimers within the PfuMoaB hexamer are mainly facilitated by residues of α3- and α4-helices (Figure 3A). Residues Ile59, Leu62, Ile63, Phe66 and Ile69 in α3-helix and Leu97 in α4-helix form the central hydrophobic core of the trimer-trimer interface, which is reinforced by two symmetrical salt bridge pairs: i) Lys70 and Glu67; ii) Lys58 and Asp99 (Figure 3A). Thus, hydrophobic residues of α3-helix were identified as key residues for the association of two trimers within the hexamer. These residues are not conserved between homologous proteins from different organisms and positioned distantly from the active site (Figure 3A, Figure 4). Thus, they represent a suitable target for mutagenesis to interfere with the oligomerization of PfuMoaB.

### Structure-guided Mutagenesis of PfuMoaB

In order to disrupt the interaction between two trimers in the PfuMoaB hexamer, first, three central hydrophobic residues of α3-helix - Ile59, Ile63 and Phe66 - were exchanged against positively charged arginines (Figure 3B). The resulting variant PfuMoaB I59R/I63R/F66R was expressed in *E. coli* as N-terminally His-tagged protein and affinity purified with comparable yields to PfuMoaB wild-type (PfuMoaB-WT), however an increased tendency to precipitation was observed. Subsequent size exclusion chromatography showed that the I59R/I63R/F66R variant was purified as a heterogeneous mixture of diverse oligomeric states (Figure S1). This finding suggests that mutagenesis of the selected residues was either not sufficient to disrupt the respective hexamer interface interactions, or that the exposure of hydrophobic residues derived from α3-helix caused non-specific protein-protein interactions.

Next, the entire α3-helix of PfuMoaB-WT was exchanged against the corresponding helix of the trimeric *E. coli* MogA (EcoMogA) homologue with two exceptions. In the PfuMoaB-WT structure, Ala64 and Ala68 are positioned at the opposite side of the hexamer interface. Their replacement against the corresponding EcoMogA Thr and Leu residues, respectively, would result in the introduction of two bulky residues and, consequently, would lead to steric clashes. Considering the fact that alanine has high helix-forming propensities, Ala64 and Ala68 of PfuMoaB were not subjected to mutagenesis. The generated variant – PfuMoaB-H3– contained an 11-residues substitution (Figure 3B), which resulted in the introduction of charged residues at the hydrophobic interface between trimers and disruption of two hexamer-stabilizing salt bridge pairs: Lys58-Asp99 and Glu63-Lys70.

### Biochemical Characterization of the PfuMoaB-H3 Variant

**Figure 5:**
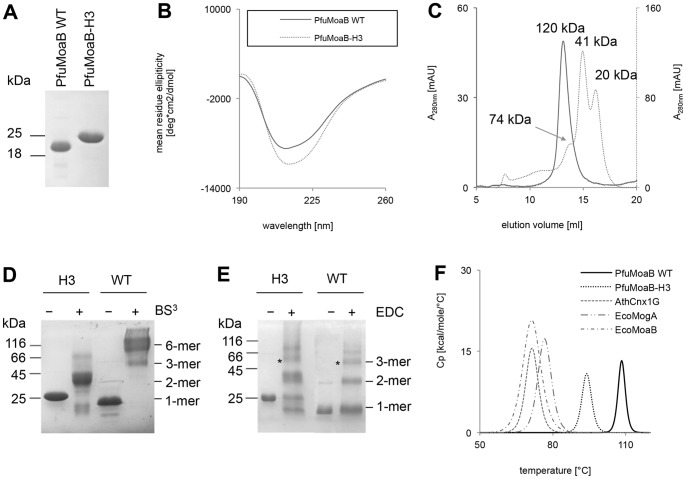
Biochemical characterization of the PfuMoaB-H3 variant in comparison to PfuMoaB-WT. (A) 15% Coomassie-Blue-stained SDS polyacrylamide gel showing 200 pmol of Ni-NTA-purified PfuMoaB-WT and PfuMoaB-H3. (B) Far-UV CD spectra of Ni-NTA purified PfuMoaB-WT (solid line) and PfuMoaB-H3 (dotted line). (C) Size exclusion chromatography of Ni-NTA purified PfuMoaB-WT and PfuMoaB-H3. 5 nmol of WT and 10 nm of PfuMoaB-H3 were applied on a Superdex 200 10/300 column. Peaks referring to the different oligomerization states of both proteins are labelled. Molecular masses were determined using protein standards. Elution of PfuMoaB-WT is shown as solid line, the PfuMoaB-H3 variant as dotted line. (D–E) SDS-PAGE of cross-linked PfuMoaB-WT and PfuMoaB-H3 with BS^3^ (D) and EDC (E). Samples without addition of cross-linkers were used as control (“–”). Observed oligomeric forms of both proteins are labelled. The cross-linked protein bands with a size corresponding to the trimers (designated with *) were further subjected to mass spectrometry analysis. (F) Differential scanning calorimetry of MPT-adenylyl-transferases. Melting curves of PfuMoaB-WT, PfuMoaB-H3, EcoMogA, EcoMoaB and AthCnx1G recorded by DSC. The maximum of each peak represents the respective *T*_m_ value. Average *T*_m_ values for each protein are summarized in the Table 2. Measurements were performed in duplicate for each experiment.

The PfuMoaB-H3 variant was purified to homogeneity in the same way as PfuMoaB-WT (Figure 5A). Interestingly, PfuMoaB-H3 was observed to migrate slower in the SDS-PAGE than expected with a corresponding band of 25 kDa in size, while its predicted mass of 19,887.68 Da is similar to that of PfuMoaB-WT (19,971.95 Da). In order to confirm the accurate mass of PfuMoaB-H3, purified PfuMoaB-WT and PfuMoaB-H3 were subjected to mass spectrometry analysis, which detected masses of 19,872 Da and 19,888 Da, respectively (data not shown). Therefore, the observed altered gel mobility of PfuMoaB-H3 could be a result of an altered mass-to-charge ratio within the SDS-PAGE due to reduced SDS binding to the protein as demonstrated for other proteins [28], [29].

In order to investigate protein folding, purified PfuMoaB-H3 and PfuMoaB-WT were subjected to circular dichroism (CD) spectroscopy. The far-UV CD spectrum of the H3 variant resembled that of PfuMoaB-WT in shape with a slight signal increase in the range of 210 to 225 nm (Figure 5B), where both α-helical and β-sheet elements have their negative maxima [30]. Possibly, the introduced changes caused partial transformation of some loop-containing regions of the protein variant into folded elements.

Next, the oligomerization state of the PfuMoaB-H3 variant was determined by size exclusion chromatography. Three major peaks with molecular masses of 74, 41 and 20 kDa were observed for PfuMoaB-H3, which correspond to trimers, dimers and monomers, respectively (Figure 5C). This finding confirmed on one hand the successful dissociation of the hexamer in PfuMoaB-H3 (as compared to PfuMoaB-WT), but on the other hand suggested weakening of interactions within the PfuMoaB-H3 trimer, giving rise to the presence of monomers and dimers in addition to trimers.

In order to demonstrate that the observed trimeric form of the PfuMoaB-H3 variant was not a random assembly of monomers, but rather the result of monomer-monomer interactions similar to that at the trimer interface of PfuMoaB-WT (Figure 2B–C), we performed chemical cross-linking of both proteins using two different reagents: (i) bis(sulfosuccinimidyl)suberate (BS^3^), which enables formation of bonds between primary amines and (ii) ethyl-3-[3-dimethylaminopropyl]carbodiimide hydrochloride (EDC), which couples carboxyl groups to primary amines. Cross-linked PfuMoaB-H3 and PfuMoaB-WT were analysed by SDS-PAGE. Thereby, PfuMoaB-WT was observed mainly as hexamer following BS^3^-cross-linking (Figure 5D), or - as trimer and dimer upon EDC treatment (Figure 5E). In contrast, the PfuMoaB-H3 variant was found to form only dimers and, to a lesser extent, trimers, when cross-linked with BS^3^ or EDC, thus confirming the results of size exclusion chromatography.

To identify the respective contact sites within the EDC-cross-linked trimers, protein bands of PfuMoaB-WT and the PfuMoaB-H3 variant were isolated from the SDS gel (Figure 5E) and subjected to peptide mass finger printing. The majority of the identified peptide sequences represented cross-links within monomers (Table 1). Similar quantities of peptides corresponding to surface-exposed intra-molecular salt-bridges were detected for WT and the PfuMoaB-H3 variant demonstrating that monomers of both proteins exhibit similar folds as already suggested by CD spectroscopy. In contrast, peptides embracing Arg109 and Glu106, which form a salt-bridge at the trimer interface, were absent for both PfuMoaB-WT and PfuMoaB-H3 (Table 1), probably due to the deeply buried position of Arg109 (Figure 2B). Peptides comprising residues of another trimer interface salt-bridge between Glu92 and Lys100 were found only in PfuMoaB-H3 (Table 1). In the crystal structure of PfuMoaB-WT both Glu92 and Lys100 are buried within the hexamer. Therefore, our results suggest a solvent-exposed nature of the inter-trimer cluster Glu92-Arg124-Lys100 in the PfuMoaB-H3 variant, which is in agreement with a dissociation of the hexamer along the two-fold symmetry axis. The identification of the characteristic inter-subunit interactions of the trimer in the cross-linked PfuMoaB-H3 supports its ability to oligomerize using similar contact sites as WT PfuMoaB and thus confirms the existence of WT-like trimers in solutions. However, in PfuMoaB-H3 also additional cross-linked peptides were observed, which were not detected in the PfuMoaB-WT (Table S2). This suggests an increased accessibility of corresponding surface residues, due to a higher dissociation of the trimer as detected in size exclusion chromatography.

**Table 1:** Analysis of the EDC cross-linked trimers of PfuMoaB-WT and the PfuMoaB-H3 variant by peptide mass fingerprinting.

Cross-link type	Cross-linked peptides^2^	Cross-linked residues^2^	N° PSMs^1^	
			WT	H3
**intra-subunit**	SYEEVGYATVLTR - AKSYEEVGYATVLTR	E114-K111	6	6
	SGPLIIEELSK - TGLEIIKSEVFHILK	E41-K157	7	9
	TGLEIIK - AKSYEEVGYATVLTR	E154-K111	3	2
	TFKFGVITVSDK - LGEHVYYK	K15-E47	4	0
	DITIESIKPLFDK - AKSYEEVGYATVLTR	E92-R124	0	8
	DITIESIKPLFDK - SYEEVGYATVLTR	E92-R124	0	3
	DITIESIKPLFDKELSFGEVFR - SYEEVGYATVLTR	E92-R124	0	2
**inter-subunit**	DITIESIKPLFDK - DITIESIKPLFDK	E92-K100	0	6

1 trypsinized cross-linked peptides were identified by LC-MS/MS; their sequences and number of peptide-spectrum matches (PSMs) are shown.

2 residues, which are suggested to be cross-linked by EDC are shown in a separate column and underlined in the respective peptide sequences.

### Thermal Stability of the PfuMoaB-H3 Variant

To probe the impact of oligomerization on thermal stability of PfuMoaB, the melting temperatures (*T*_m_) of both PfuMoaB-WT and the PfuMoaB-H3 variant were determined by differential scanning calorimetry (DSC). Thermal melting of PfuMoaB-WT and PfuMoaB-H3 followed single endothermic transitions, with a *T*_m_ of 108.6°C and 93.3°C, respectively (Figure 5F), suggesting a simultaneous dissociation of the oligomers and denaturation of the protomers. The reduction of the apparent melting temperature in PfuMoaB-H3 by 15°C as compared to PfuMoaB-WT demonstrates the significant contribution of the trimer-trimer interface in thermal stability of PfuMoaB.

For comparison, *T*_m_ values of mesophilic homologues of PfuMoaB were determined: trimeric *E. coli* MogA (EcoMogA) and the G-domain of *A. thaliana* Cnx1 (AthCnx1G), as well as hexameric *E. coli* MoaB (EcoMoaB). They were expressed as His-tagged fusions in *E. coli* and purified to homogeneity (Figure S2A). Following the determination of their physiological oligomeric state by size exclusion chromatography (Figure S2B), DSC analysis revealed *T*_m_ values of 76.1, 69.6 and 71°C for EcoMogA, EcoMoaB and AthCnx1G, respectively (Figure 5F). Thus, the observed melting points of the PfuMoaB mesophilic homologues were relatively close to each other and significantly lower than that of the PfuMoaB-H3 variant (Table 2). Interestingly, hexameric EcoMoaB showed an even lower melting temperature than trimeric EcoMogA. Given the remaining difference in the melting temperatures between PfuMoaB-H3 and its mesophilic homologues, we conclude that additional factors contribute to the thermal stability of PfuMoaB.

**Table 2:** Melting temperatures (*T*_m_) of MPT adenylyl-transferases from different organism measured by differential scanning calorimetry (DSC).

Protein^1^	*T*_m_ [°C]^2^
PfuMoaB-WT	108.6±0.5
PfuMoaB-H3	93.3±0.5
EcoMoaB	69.6±0.8
EcoMogA	76.1±0.3
AthCnx1G	71.0±0.3
RnoGephG [61]	72.4

1 proteins from following organisms were used: PfuMoaB, *Pyrococcus furiosus*; EcoMoaB and EcoMogA, *Escherichia coli*; AthCnx1G, *Arabidopsis thaliana*; RnoGeph, *Rattus norvegicus*.

2 average *T*_m_ values derived from two independent DSC experiments.

### Functional Characterization of the PfuMoaB-H3 Variant

To understand the contribution of hexamer formation to the PfuMoaB function, the activity of the purified PfuMoaB-H3 variant and PfuMoaB-WT was analysed by monitoring the conversion of MPT into MPT-AMP at different temperatures. MPT was first synthesised *in vitro* using purified *E. coli* cPMP and MPT synthase subunits MoaD and MoaE in the presence of PfuMoaB-WT or PfuMoaB-H3 and was subsequently adenylylated through addition of ATP and MgCl_2._

**Figure 6:**
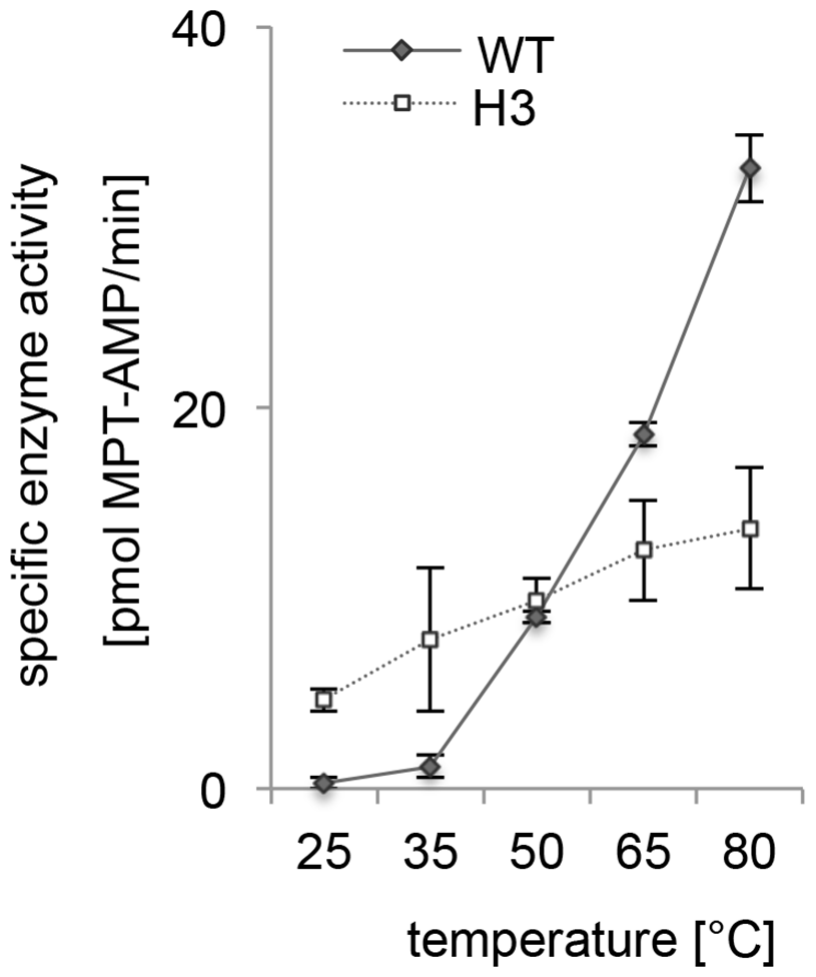
*In vitro* adenylylation of MPT by PfuMoaB-WT and PfuMoaB-H3 variant. Adenylylation rates were determined for both proteins at 25, 35, 50, 65 and 80°C by monitoring formation of MTP-AMP over time. Initial velocities of PfuMoaB-WT and the H3 variant at different temperatures are depicted as solid and dotted lines, respectively. Error bars represent the standard deviation of data obtained in at least two independent experiments.

PfuMoaB-WT showed a temperature-dependent increase in MPT-AMP synthesis with a minor decrease at longer incubation times at 80°C (Figure S3E). While at 50°C both WT and H3 exhibited similar activities (Figure S3C), at high temperatures (65°C and 80°C) a significant activity loss of PfuMoaB-H3 was observed (Figure S3D–E). Interestingly, at moderate temperatures of 25°C and 35°C, the H3 variant was 16-fold and 7-fold, respectively, more active than PfuMoaB-WT (Figure 6, Figure S3A–B). First, these results demonstrate that PfuMoaB-H3 remained catalytically potent, which supports our previous observation that the overall folding of the protomers has not been dramatically changed. Second, the decreased number of interactions in PfuMoaB-H3 following the dissociation of the hexamer, have probably enhanced the degree of conformational freedom within PfuMoaB-H3, thus leading to the improved catalytic velocity at mild temperatures.

## Discussion

In the current study the impact of hexamerization on the thermostability of the tungsten cofactor synthesising protein PfuMoaB has been investigated. Based on the crystal structure of PfuMoaB, which we determined at 2.5 Å resolution, residues of the α3-helix were shown to form the central core of the hexamerization interface. Exchange of α3-helix against the corresponding helix of the trimeric PfuMoaB homologue EcoMogA resulted in a dissociation of the PfuMoaB hexamer and a decrease of the apparent melting temperature by more than 15°C. Therefore, hexamerization of PfuMoaB is a key determinant in thermal stability of PfuMoaB. Our finding supports earlier studies that have identified increased oligomerization states in multi-subunit proteins from thermophilic organisms [31]–[33]. At this point, we cannot exclude that, in addition to the changed oligomerization, the introduction of the new a3-helix on its own has change the stability of the PfuMoaB-H3 monomer. However, given the fact that the DSC profile of PfuMoaB-H3 again shows only a single transition from the folded to the unfolded state, suggests that similar to WT, PfuMoaB-H3 denatures in a single step. Together with our cross-linking studies we conclude that the introduced a3-helix unfolds in concert with the remaining protein and does not impact the overall fold of the PfuMoaB protomer significantly. Unfortunately, we were not able to produce crystals for structural analysis of PfMoaB-H3, which in turn could have provided ultimate proof of the structural alterations in PfMoaB-H3.

The fact, that the melting temperature of PfuMoaB-H3 was still 17–23°C higher than that of its mesophilic homologues suggests besides hexamerization additional factors contributing to the thermal stability of PfuMoaB. Various studies identified structural determinants such as increased number of hydrogen bonds [34], [35] and ionic interactions [36], [37], shortening of loop regions [38], [39], more extensive hydrophobic interactions [40], [41], as well as decreased content of thermolabile residues [42], [43] and increased proline content [44], [45] as mechanisms to increase stability of proteins. Furthermore, it has been shown that thermostable proteins belonging to the same family can develop different strategies to adapt to high temperatures [46]–[48]. The latter will explain the presence of non-hexameric MPT-adenylyl-transferases of the MogA-subfamily in some thermophilic organisms, e.g. bacteria *Thermus thermophilus* (TthMogA) and *Aquiefex aeolicus* (AaeMogA). As an increase in the number of stabilizing inter-molecular interactions via hexamerization is not available for those enzymes, other mechanisms must contribute to thermal stability. We compared crystal structures and polypeptide sequences of trimeric TthMogA and AaeMogA with hexameric PfuMoaB and observed an increased number of intramolecular salt bridges and an increased content of proline residues in both TthMogA and AaeMogA (table S3). Furthermore, a remarkably high number of salt bridges at the trimerization interface of AaeMogA was found (table S3). These findings suggest that stabilization of the individual subunits and the trimer are the key mechanisms in MogA-type of MPT-adenylyl-transferases to adapt to elevated temperatures.

The impact of PfuMoaB mutagenesis and the resulting change of the oligomerization state on catalytic activity of the PfuMoaB-H3 variant was investigated at different temperatures. Significant reduction of the PfuMoaB-H3 activity was observed at temperatures much lower than its apparent melting point, indicating that the loss of activity was initiated before irreversible denaturation of the protein. Similar findings were reported for 3-phosphoglycerate kinase from thermophilic *Thermoanaerobacter sp. Rt8.G4* and mesophilic *Zymomonas mobilis* [49], 2-keto-3- deoxygluconate aldolase from *S. solfataricus* [50] and citrate synthase from *Thermoplasma acidophilum* [51]. One can assume that increased conformational flexibility of the active site enhances its susceptibility to unfolding upon increase of temperature and therefore, during the process of thermal denaturation, the active site is one of the first regions to adapt a partially unfolded conformation. Consequently, the loss of enzyme activity is seen at lower temperatures than irreversible unfolding of the entire protein [49].

Remarkably, at moderate temperatures of 25°C and 35°C PfuMoaB-H3 was found to be more active in comparison to the WT protein. To our knowledge, this is the first report of increased catalytic activity for a given enzyme, following the alteration of its oligomerization state. Activity and conformational flexibility of enzymes are tightly interconnected thus reflecting their dynamic nature. During catalysis, enzymes undergo sequential conformational changes to enable substrate binding and stabilization of the transition state, followed by product formation and release [52]–[54]. Increased stability of proteins from thermophilic organisms inevitably leads to an increase in rigidity, which in turn affects their conformational flexibility. For 3-isopropylmalate dehydrogenase from *T. thermophilus*, a lower activity of the enzyme at room temperature in comparison to its mesophilic homologue from *E. coli* was attributed to restricted conformational movements at sub-optimal temperatures of the thermophilic protein [55]. In contrast, at temperatures close to the optimal activity (*T*_opt_) the conformational flexibility of the enzyme significantly increased [55]. Similar findings were reported for thermostable glyceraldehyde-3-phosphate dehydrogenase from *T. maritima* [56].

**Figure 7:**
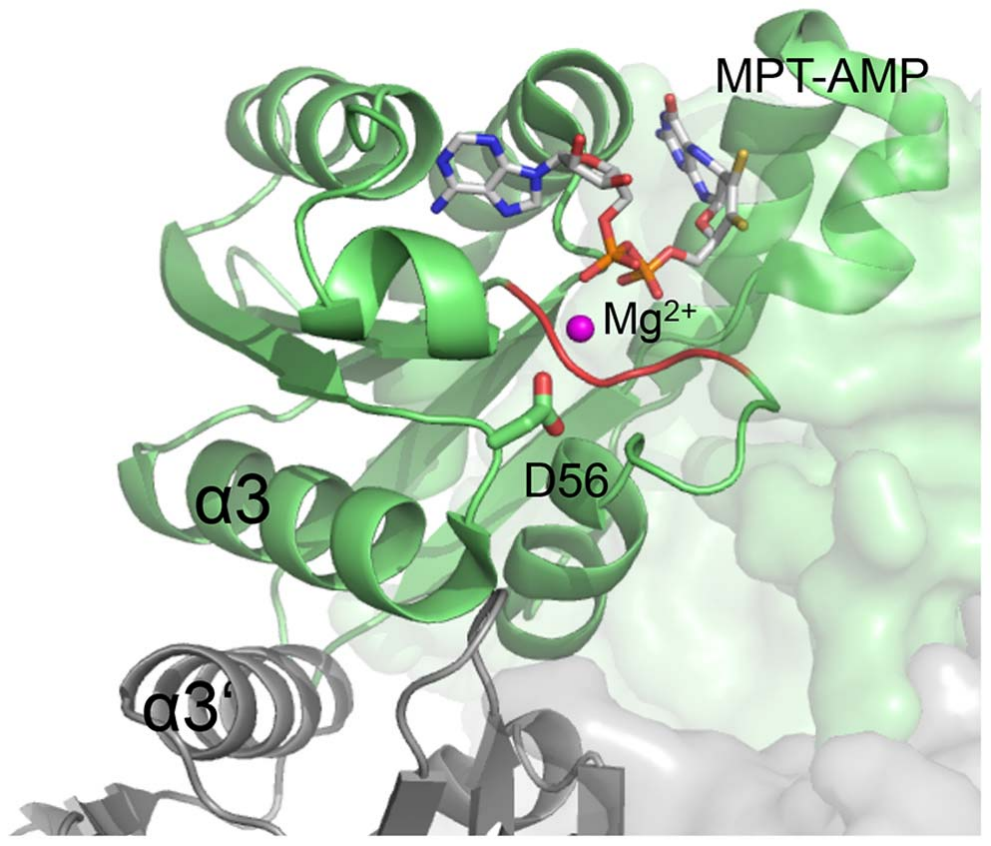
Active site of PfuMoaB-WT. Two PfuMoaB subunits at the hexamerization interface are shown as ribbon in green and grey, respectively. The conserved Asp56 residue coordinating Mg^2+^ (pink) is shown in sticks. MPT-AMP in the active site is derived from a superimposition with the structure of the PfuMoaB homologue *A. thaliana* Cnx1G (1UUY). The Mg^2+^-ion derived from a superimposition with the homologues sub-domain 3 of *E. coli* MoeA (1FC5) [6], [63].

Disruption of major stabilizing interactions at the interface of the PfuMoaB hexamer might have provided an additional degree of freedom to residues in individual subunits and thus, increased their conformation flexibility and resulted in much higher activity at moderate temperatures. It is important to note that a3-helix extends into a loop hosting the highly conserved Asp56 (Figure 7), which was previously found to be crucial for the catalytic function of the plant homologue Cnx1G [57]. Later we could demonstrate that the corresponding Asp515 of Cnx1G is involved in the coordination of Mg^2+^-ion during ATP-dependent MPT adenylylation [6], [7]. Consequently, once can assume that Asp56 in PfuMoaB undergoes conformational changes during catalysis. Therefore, increased conformational flexibility, introduced by the exchange of a3-helix, has probably released the structural constrain present in the hexameric PfuMoaB thus explaining the gain in catalytic velocity at lower temperature.

Remarkably, Kananujia *et al.* showed by molecular-docking simulations that MogA proteins bind MPT stronger than MoaB proteins [58]. Together with the observed increased activity of PfuMoaB-H3, these results might suggest that lower oligomeric forms of MPT-adenylyl-transferases are more efficient at moderate temperatures. This might explain why only representatives of the MogA sub-family, but not of the MoaB-sub-family, are found today in eukaryotes (Figure S4).

Interestingly, MogA and MoaB homologues are not equally distributed in prokaryotes. In Bacteria, species with only MogA (*e. g. A. aeolicus*, *Clostridium botulinum*), only MoaB (*e. g. B. cereus*, *Streptomyces avermitilis*, *Vibrio vulnificus*), or both homologues (*e. g. E. coli, Azorhizobium caulinodans, Bordetella bronchiseptica*) are detected. In contrast, search of the non-redundant protein sequence database in Archaea revealed only three organisms containing MogA proteins, which belong to the phylogenetically close orders of *Desulforococcales* (*Pyrolobus fumarii* and *Aeropyrum pernix*) and *Acidolobales* (*Acidilobus saccharovorans*). Domination of MoaB in Archaea might indicate that MogA is unable to fulfil yet unknown functions of MoaB in archaeal organisms. In *E. coli*, enzymes catalysing the last steps of Moco biosynthesis (MogA, MoeA, MobA and MobB) were found to form a transient multi-protein complex *in vivo*, most probably to protect unstable reaction intermediates, and to secure targeting of the cofactor to the corresponding apo-enzymes [59]. Considering the fact that MoaB forms hexamers, this oligomerization state might provide an additional surface for interactions with enzymes upstream and downstream of MPT adenylylation.

Noteworthy, EcoMoaB was found to be inactive for MPT adenylylation both *in vivo* and *in vitro* [8], [60] demonstrating that its function in Moco biosynthesis was completely taken over by the MogA homologue. Future functional characterisation of MoaB homologues from bacterial organisms without MogA might clarify whether MoaB proteins are Archaea-specific proteins unable to function in Bacteria, or whether the MoaB-type MPT-adenylyl-transferases are common for all prokaryotes, while MogA is a Bacteria-specific representative.

## Supporting Information

Figure S1 **Size exclusion chromatography (SEC) of the PfuMoaB I59R/I63R/F66R variant.** SEC elution profile of Ni-NTA purified PfuMoaB I59R/I63R/F66R variant using Superdex 200 16/60 pg column (GE Healthcare). Collected fractions (highlighted with arrows) were subsequently analysed by SDS-PAGE (shown in the SEC-chromatogram).
(DOCX)

Figure S2 **Purification of His-tagged fusion proteins EcoMogA, EcoMoaB and AthCnx1G expressed in** ***E. coli.*** (A) Coomassie-Blue-stained 15% SDS polyacrylamide gel of Ni-NTA purified proteins. (B) Size exclusion profiles of the Ni-NTA purified EcoMogA, EcoMoaB and AthCnx1G using Superdex 200 10/300 column. 10 nmol of each protein was applied. Observed molecular masses of the peaks correspond to EcoMoaB hexamers and EcoMogA and AthCnx1G trimers.
(DOCX)

Figure S3 ***In vitro*** **adenylylation of MPT by PfuMoaB-WT and the PfuMoaB-H3 variant.** Adenylylation rates were determined for both proteins at 25°C (A), 35°C (B), 50°C (C), 65°C (D) and 80°C (E) by monitoring formation of MTP-AMP in time. Adenylylation kinetics of PfuMoaB-WT and the PfuMoaB-H3 variant are depicted as solid and dotted lines, respectively. Measurements were performed in duplicate for each experiment, error bar represent the standard deviation of data obtained in two separate experiments.
(DOCX)

Figure S4 **Phylogenetic tree of MPT-adenylyl-transferases.** MPT adenylyl-transferases from following organisms are shown: *A. thaliana* (AthCnx1G), *H. sapiens* (HsaGephG), *T. thermophilus* (TthMogA), *A. aeolicus* (AaeMogA), *E. coli* (EcoMoaB and EcoMogA), *B. cereus* (BceMoaB), *S. tokodaii* (StoMoaB) and *P. furiosus* (PfuMoaB). Branch lengths are shown in per cent. Red branches - Archaea, violet - Bacteria, blue - Eukaryotes. Phylogenetic tree was prepared using web server of the Le Laboratoire d’Informatique, de Robotique et de Microélectronique of University Montpellier, France www.phylogeny.fr [64].
(DOCX)

Table S1 Collection of diffraction data and refinement statistics.
(DOCX)

Table S2 Non-specific cross-links in trimers of PfuMoaB-WT and the PfuMoaB-H3 variant identified by peptide mass fingerprinting.
(DOCX)

Table S3 Comparative analysis of *P. furiosus* MoaB, *A. aeolicus* MogA and *T. thermophilus* MogA.
(DOCX)
