# Supplementary figures and images for: Structural Basis of Thermal Stability of the Tungsten Cofactor Synthesis Protein MoaB from Pyrococcus furiosus
Source: PLoS One. 2014 Jan 20;9(1):e86030. doi: 10.1371/journal.pone.0086030 (PMC3896444; doi:10.1371/journal.pone.0086030)

**
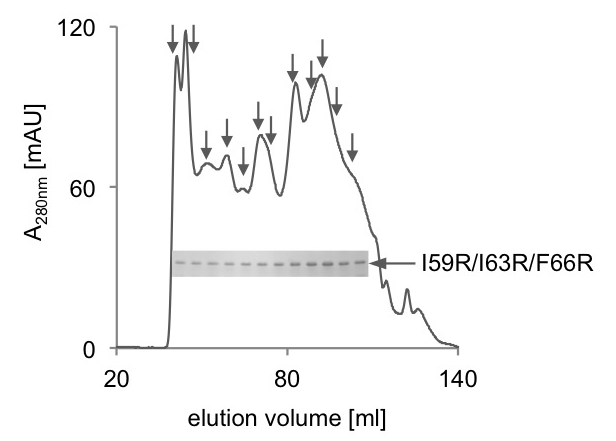
**

Figure S1. Size exclusion chromatography (SEC) of the PfuMoaB I59R/I63R/F66R variant.

Supplement: Figure S1 — Size exclusion chromatography (SEC) of the PfuMoaB I59R/I63R/F66R variant. SEC elution profile of Ni-NTA purified PfuMoaB I59R/I63R/F66R variant using Superdex 200 16/60 pg column (GE Healthcare). Collected fractions (highlighted with arrows) were subsequently analysed by SDS-PAGE (shown in the SEC-chromatogram). (DOCX) [file pone.0086030.s001.docx]

**
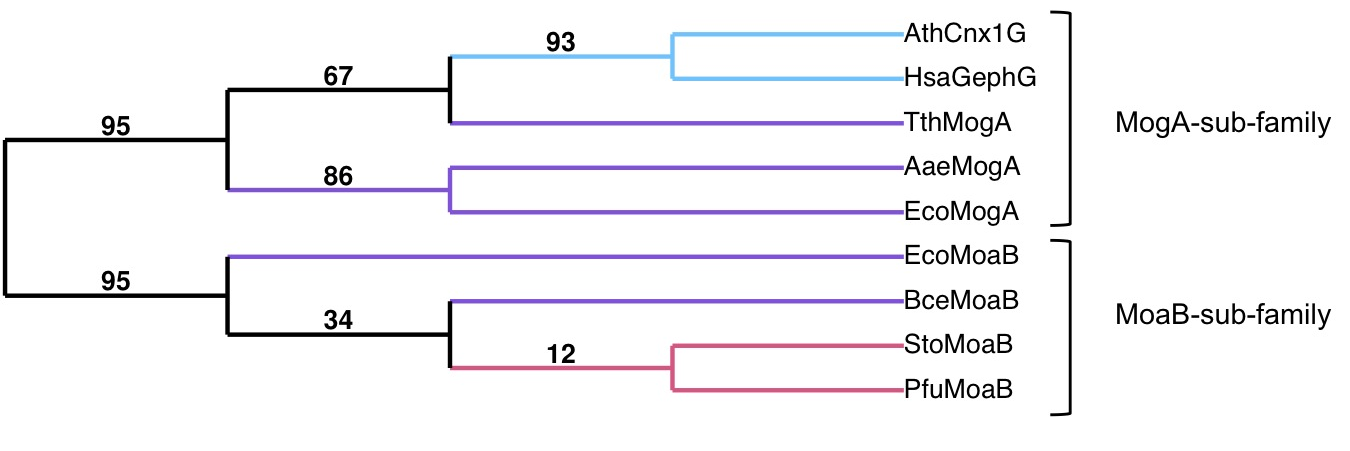
**

Figure S4. Phylogenetic tree of MPT-adenylyl-transferases.

Supplement: Figure S4 — Phylogenetic tree of MPT-adenylyl-transferases. MPT adenylyl-transferases from following organisms are shown: A. thaliana (AthCnx1G), H. sapiens (HsaGephG), T. thermophilus (TthMogA), A. aeolicus (AaeMogA), E. coli (EcoMoaB and EcoMogA), B. cereus (BceMoaB), S. tokodaii (StoMoaB) and P. furiosus (PfuMoaB). Branch lengths are shown in per cent. Red branches - Archaea, violet - Bacteria, blue - Eukaryotes. Phylogenetic tree was prepared using web server of the Le Laboratoire d’Informatique, de Robotique et de Microélectronique of University Montpellier, France www.phylogeny.fr [64]. (DOCX) [file pone.0086030.s004.docx]
