# Supplementary material for: Structural Basis of Thermal Stability of the Tungsten Cofactor Synthesis Protein MoaB from Pyrococcus furiosus
Source: PLoS One. 2014 Jan 20;9(1):e86030. doi: 10.1371/journal.pone.0086030 (PMC3896444; doi:10.1371/journal.pone.0086030)
Supplement: Figure S2 — Purification of His-tagged fusion proteins EcoMogA, EcoMoaB and AthCnx1G expressed in E. coli. (A) Coomassie-Blue-stained 15% SDS polyacrylamide gel of Ni-NTA purified proteins. (B) Size exclusion profiles of the Ni-NTA purified EcoMogA, EcoMoaB and AthCnx1G using Superdex 200 10/300 column. 10 nmol of each protein was applied. Observed molecular masses of the peaks correspond to EcoMoaB hexamers and EcoMogA and AthCnx1G trimers. (DOCX) [file pone.0086030.s002.docx]

| **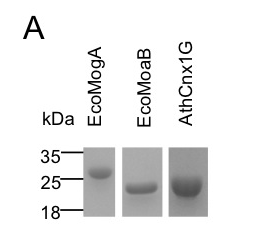** | **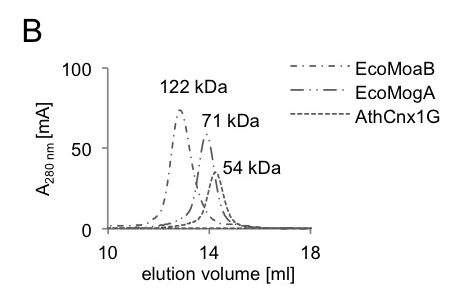** |
| --- | --- |

Figure S2. Purification of His-tagged fusion proteins EcoMogA, EcoMoaB and AthCnx1G expressed in *E. coli.*
