# Supplementary material for: Structural Basis of Thermal Stability of the Tungsten Cofactor Synthesis Protein MoaB from Pyrococcus furiosus
Source: PLoS One. 2014 Jan 20;9(1):e86030. doi: 10.1371/journal.pone.0086030 (PMC3896444; doi:10.1371/journal.pone.0086030)
Supplement: Figure S3 — In vitro adenylylation of MPT by PfuMoaB-WT and the PfuMoaB-H3 variant. Adenylylation rates were determined for both proteins at 25°C (A), 35°C (B), 50°C (C), 65°C (D) and 80°C (E) by monitoring formation of MTP-AMP in time. Adenylylation kinetics of PfuMoaB-WT and the PfuMoaB-H3 variant are depicted as solid and dotted lines, respectively. Measurements were performed in duplicate for each experiment, error bar represent the standard deviation of data obtained in two separate experiments. (DOCX) [file pone.0086030.s003.docx]

Figure S3. *In vitro* adenylylation of MPT by PfuMoaB-WT and the PfuMoaB-H3 variant.
