# Supplementary material for: Structural Basis of Thermal Stability of the Tungsten Cofactor Synthesis Protein MoaB from Pyrococcus furiosus
Source: PLoS One. 2014 Jan 20;9(1):e86030. doi: 10.1371/journal.pone.0086030 (PMC3896444; doi:10.1371/journal.pone.0086030)
Supplement: Table S1 — Collection of diffraction data and refinement statistics. (DOCX) [file pone.0086030.s005.docx]

Table S1. Collection of diffraction data and refinement statistics

| **Space group** | P3_1_21 |
| --- | --- |
| **Unit cell** |  |
| a, b, c, (Å) | 125.9, 125.9, 73.4 |
| α, β, γ (°) | 90, 90, 120 |
| **Data collection** |  |
| Resolution range, (Å) | 2.50-2.64 |
| Wavelength, (Å) | 0.918 |
| Total reflections | 292771 (42337) |
| Reflections unique | 23557(3383) |
| Completeness, (%) | 100 (100) |
| Mean I/sd(I) | 18.0 (3.7) |
| Rmerge (%) | 11.1 (8.1) |
| Mosaicity, (°) | 0.47 |
| **Refinement** |  |
| Resolution range, (Å) | 41.22-2.50 (2.50-2.56) |
| N° of reflections | 22319 |
| *R*-work | 0.18 |
| *R*-free | 0.24 |
| DPI, (Å) | 0.233 |
| Root mean square deviation |  |
| bonds length, (Å) | 0.02 |
| bonds angles, (°) | 1.93 |
| N° of protein atoms | 3691 |
| N° of water molecules | 116 |
| SO_4_^2-^ | 3 |
| Average B-factor (Wilson), (Å^2^) | 43.65 |
| Ramachandran plot, (%) |  |
| in preferred regions | 95.99 |
| in allowed regions | 4.01 |
| in disallowed regions | 0 |
