# Supplementary material for: Structural Basis of Thermal Stability of the Tungsten Cofactor Synthesis Protein MoaB from Pyrococcus furiosus
Source: PLoS One. 2014 Jan 20;9(1):e86030. doi: 10.1371/journal.pone.0086030 (PMC3896444; doi:10.1371/journal.pone.0086030)
Supplement: Table S2 — Non-specific cross-links in trimers of PfuMoaB-WT and the PfuMoaB-H3 variant identified by peptide mass fingerprinting. (DOCX) [file pone.0086030.s006.docx]

Table S2. Non-specific cross-links in trimers of PfuMoaB-WT and the PfuMoaB-H3 variant identified by peptide mass fingerprinting

| **Identified peptides** | **N° PSMs^1^** | |
| --- | --- | --- |
|  | **WT** | **H3** |
| FGVITVSDKGAK - HARE | 4 | 0 |
| FGVITVSDKGAK - GQER | 2 | 2 |
| EAPK - AKSYEEVGYATVLTR | 1 | 0 |
| EAPKTFK - SYEEVGYATVLTR | 0 | 2 |
| GQER - AKSYEEVGYATVLTR | 0 | 6 |
| SEVFHILKHAR - SYEEVGYATVLTR | 0 | 7 |
| HARE - AKSYEEVGYATVLTR | 0 | 2 |
| FGVITVSDKGAK - ELSFGEVFR | 0 | 7 |
| FGVITVSDKGAK - SYEEVGYATVLTR | 0 | 4 |
| FGVITVSDK - AKSYEEVGYATVLTR | 0 | 2 |
| TFKFGVITVSDK - SYEEVGYATVLTR | 0 | 2 |
| FGVITVSDKGAK - DITIESIKPLFDK | 0 | 7 |
| FGVITVSDKGAK - DITIESIKPLFDKELSFGEVFR | 0 | 5 |
| SEVFHILKHAR - ELSFGEVFR | 0 | 6 |
| SGPLIIEELSKLGEHVYYK - SYEEVGYATVLTR | 0 | 2 |

^1^trypsinized cross-linked peptides were identified by LC-MS/MS; their sequences and number of peptide-spectrum matches (PSMs) are shown.
