# Supplementary material for: Structural Basis of Thermal Stability of the Tungsten Cofactor Synthesis Protein MoaB from Pyrococcus furiosus
Source: PLoS One. 2014 Jan 20;9(1):e86030. doi: 10.1371/journal.pone.0086030 (PMC3896444; doi:10.1371/journal.pone.0086030)
Supplement: Table S3 — Comparative analysis of P. furiosus MoaB, A. aeolicus MogA and T. thermophilus MogA. (DOCX) [file pone.0086030.s007.docx]

**Table S3. Comparative analysis of *P. furiosus* MoaB, *A. aeolicus* MogA and *T. thermophilus* MogA**

| **Domain** | Archaea | Bacteria | Bacteria |
| --- | --- | --- | --- |
| **Species** | *Pyrococcus furiosus* | *Aquifex aeolicus* | *Thermus thermophilus* |
| **Max. living temperature, °C** | 103 | 95 | 85 |
| **MPT-adenylyl-transferase** | MoaB | MogA | MogA |
| **Oligomerization state** | hexamer | trimer | trimer |
| **Crystal structure (RCSB PDB)** | 4LHB | 3MCI | 3MCH |
| **Resolution, Å** | 2.6 | 1.9 | 1.6 |
| **N° of residues** | 169 | 178 | 164 |
| **N° of residues in structure** | 159 | 177 | 159 |
|  |  |  |  |
| **Proline content^1^, %** | 2.96 | 7.30 | 7.93 |
|  |  |  |  |
| **Non α-helical, β-sheet regions^2^, %** | 22.0 | 33.3 | 28.3 |
|  |  |  |  |
| **N° of hydrogen bonds^3^** |  |  |  |
| within monomer | 271 | 322 | 296 |
| at trimerization interface | 80 | 31 | 31 |
| at hexamerization interface | 55 | - | - |
|  |  |  |  |
| **N° of salt bridges^3^** |  |  |  |
| within monomer | 5 | 11 | 10 |
| at trimerization interface | 6 | 38 | 4 |
| at hexamerization interface | 12 | - | - |
|  |  |  |  |
| **Interface area^4^, Å** |  |  |  |
| trimerization interface | 1800 | 1476 | 1566 |
| hexamerization interface | 1938 | - | - |
|  |  |  |  |
| **Non-polar interface area^5^, Å** |  |  |  |
| trimerization interface | 1094 | 1098 | 1106 |
| hexamerization interface | 1297 | - | - |

^1^calculated based on the number of residues in sequence.

^2^calculated based on the number of residues in structure.

^3^values given for the interfaces are totals for all subunits of the respective interface.

^4^total interface area determined for two subunits involved in the interaction at the corresponding interface.

^5^total non-polar interface area determined for two subunits involved in the interaction at the corresponding interface.
